# Radiation-Stimulated Translocation of CD166 and CRYAB to the Endothelial Surface Provides Potential Vascular Targets on Irradiated Brain Arteriovenous Malformations

**DOI:** 10.3390/ijms20235830

**Published:** 2019-11-20

**Authors:** Lucinda S. McRobb, Matthew J. McKay, Andrew J. Gauden, Vivienne S. Lee, Sinduja Subramanian, Santhosh George Thomas, Markus K. H. Wiedmann, Vaughan Moutrie, Michael Grace, Zhenjun Zhao, Mark P. Molloy, Marcus A. Stoodley

**Affiliations:** 1 Department of Clinical Medicine, Macquarie University, Sydney 2109, Australiamarcus.stoodley@mq.edu.au (M.A.S.); 2 Australian Proteome Analysis Facility, Department of Molecular Sciences, Macquarie University, Sydney 2109, Australia; 3 Genesis Cancer Care, Macquarie University Hospital, Sydney 2109, Australia; 4 Kolling Institute, Northern Clinical School, University of Sydney, Sydney 2065, Australia

**Keywords:** brain arteriovenous malformation, endothelial cells, ionizing radiation, proteomics, stereotactic radiosurgery, vascular targeting

## Abstract

Vascular targeting with pro-thrombotic antibody-conjugates is a promising biological treatment for brain arteriovenous malformations (bAVMs). However, targeted drug delivery relies on the identification of unique or overexpressed markers on the surface of a target cell. In the absence of inherent biological markers, stereotactic radiosurgery may be used to prime induction of site-specific and targetable molecular changes on the endothelial surface. To investigate lumen-accessible, endothelial targets induced by radiation, we combined Gamma knife surgery in an AVM animal model with in vivo biotin-labeling and comparative proteomics. Two proteins, αB-crystallin (CRYAB)—a small heat shock protein that normally acts as an intracellular chaperone to misfolded proteins—and activated leukocyte cell adhesion molecule CD166, were further validated for endothelial surface expression after irradiation. Immunostaining of endothelial cells in vitro and rat AVM tissue ex vivo confirmed de novo induction of CRYAB following irradiation (20 Gy). Western analysis demonstrated that CRYAB accumulated intracellularly as a 20 kDa monomer, but, at the cell surface, a novel 65 kDa protein was observed, suggesting radiation stimulates translocation of an atypical CRYAB isoform. In contrast, CD166 had relatively high expression in non-irradiated cells, localized predominantly to the lateral surfaces. Radiation increased CD166 surface exposure by inducing translocation from intercellular junctions to the apical surface without significantly altering total protein levels. These findings reinforce the dynamic molecular changes induced by radiation exposure, particularly at the cell surface, and support further investigation of radiation as a priming mechanism and these molecules as putative targets for focused drug delivery in irradiated tissue.

## 1. Introduction

Brain arteriovenous malformations (AVMs) are vascular abnormalities characterized by direct artery to vein connections that shunt high-flow blood across the brain [1]. They are a significant cause of intracerebral hemorrhage, disability and death, in children and young adults [2]. Traditional approaches to AVM treatment include surgical removal, endovascular embolization, or radiosurgery, however, all have limitations and, currently, approximately one third of AVM patients remain untreatable. We have proposed that vascular targeting may be useful as an alternative treatment approach for brain AVMs. Vascular targeting was first described in cancer therapy to deliver thrombotic agents for tumor vessel destruction that capitalizes on the inherent molecular differences between tumor endothelium and normal, healthy endothelium that can be used to target agents selectively to the site of disease [3,4]. To date, no inherent, discriminatory vascular markers have been identified on human AVMs, hence we proposed that a combination of focused radiosurgical priming and vascular targeting may be an effective approach for AVM cure [5,6,7,8,9,10,11]. Radiation has previously been used to enhance target expression on tumor vasculature [12,13].

We recently utilized a technique of biotin-labeling to specifically tag and enrich endothelial surface proteins using a modified biotin that is impermeable to the cell membrane. Streptavidin enrichment of the biotinylated surface proteins prior to mass spectrometry and proteomic analysis allowed us to examine the surface proteome of irradiated endothelial cells in vitro and in vivo in order to discover putative molecular targets [5,14]. Several typically intracellular proteins that undergo an unconventional surface translocation in response to radiation were identified using this approach. The mechanisms and biological functions of these translocations remain unclear, but these findings contribute to our understanding of radiation effects on the vasculature, and, further, provide a set of novel radiation biomarkers for further investigation as putative target molecules for drug delivery. In this report, we performed further proteomic screening of radiation-stimulated surface molecules in an AVM animal model [15,16] and chose two proteins for additional examination and validation: activated leukocyte adhesion molecule (ALCAM or CD166) and αB-crystallin (CRYAB).

CD166 is a membrane glycoprotein of the immunoglobulin family that forms adhesive intercellular complexes in epithelial or endothelial layers, but also plays a role in transendothelial monocyte migration [17,18]. It has previously been investigated as a putative target in prostate cancer but has not previously been explored in any context as a radiation marker [19]. In contrast, CRYAB is a member of the small heat shock family of proteins that can act as an intracellular chaperone to misfolded proteins in response to a variety of stresses, including radiation [20,21], but can also play a protective role when excreted in exosomes [22]. Here, we further characterized the association of these targets with the luminal endothelial surface after radiation. Our findings support the observation that radiation drives both canonical and non-canonical transport pathways regulating subcellular protein localization and that focused radiosurgery may be a source of “neo-antigens” suitable for ligand-directed vascular targeting.

## 2. Results

### 2.1. LC-MS/MS and SWATH Analysis

Using a previously established method, we treated a rat model of AVM with Gamma Knife surgery and used in vivo biotin-labeling with a membrane-impermeable form of biotin to specifically label the luminal surface proteins [5]. Proteins were then extracted from the AVM tissue and the biotin-tagged proteins enriched by streptavidin pull-down. Due to the limiting protein amounts, LC-MS/MS was performed on enriched extracts from pooled samples (*n* = 4) at each time point. This enabled detection of 280 proteins, which were used to form a spectral library for SWATH-MS analysis and the quantitative comparisons of irradiated and non-irradiated AVM extracts [23]. Proteins were sorted in order of highest to lowest fold-change for day 21, and the corresponding fold-change values for day 3 and day 7 were assembled alongside (Table S1). Due to the need to pool samples, analysis focused on proteins that demonstrated a consistent fold-change of at least 1.4 across all three time points to increase the confidence in each (Table 1), rather than assessing those with the highest total expression at a single time point. The presence of the mitochondrial protein PDCE2 (pyruvate dehydrogenase complex subunit E2) in the surface extracts was consistent with our previous findings [5], further validating the utility of this method, however, this was not further examined in this study. Suitable commercial antibodies were not available for the two solute carrier proteins. Hence, CD166 and CRYAB were selected for further study to validate their regulation at the cell surface in response to radiation.

### 2.2. Radiation Stimulates Up-Regulation of CRYAB, but not CD166 Expression, in the Vascular Endothelium

**Figure 1:**
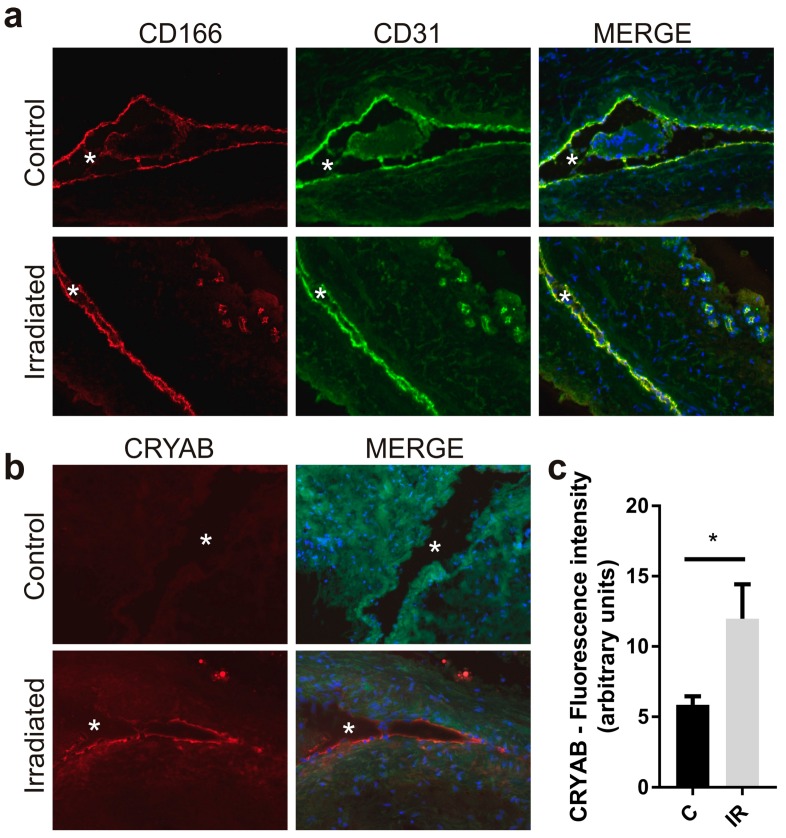
Immunohistochemical localization of CD166 and CRYAB in rat arteriovenous malformations (AVM). The surgically created AVM was excised 3 days after Gamma Knife or sham treatment and frozen for cryosectioning. Representative images of immunostained AVM vessels in the central nidus stained with antibodies targeting CD166 (**a**) or CRYAB (**b**). Target protein (AF647, red); CD166 sections were co-stained with endothelial marker CD31 (AF488, green); CRYAB sections show elevated background autofluorescence (green, 488 nm em) to outline the vessel wall; nuclei were stained with DAPI (blue). Original magnification, 200×. Asterisk indicates vessel lumen. (**c**) Mean intensity of fluorescence for CRYAB-stained sections, sham control (C) or irradiated (IR). Data represent mean fluorescence intensity ± SEM of CRYAB-stained sections from three independent animals per treatment group. Unpaired Student’s *t*-test, * *p* < 0.05.

Total levels of CRYAB and CD166 expression were examined in the vascular wall of the model AVM using immunostaining of ex vivo sections of rat AVM vessels taken 3 days after sham or Gamma Knife treatment (Figure 1). CD166 was localized specifically to the endothelium of both small and large vessels, co-localizing with the CD31 endothelial marker (Figure 1a). High basal levels of expression were observed for CD166 in the endothelium of both non-irradiated and irradiated vessels. There was no significant increase observed in response to Gamma Knife treatment. In contrast, CRYAB was absent at the endothelium in non-irradiated AVMs but, in response to radiation, there was a consistent and significant increase in the intensity of endothelial CRYAB staining (2-fold, *p* < 0.05) (Figure 1b,c).

### 2.3. Radiation Alters Subcellular Localization of CD166 and CRYAB

**Figure 2:**
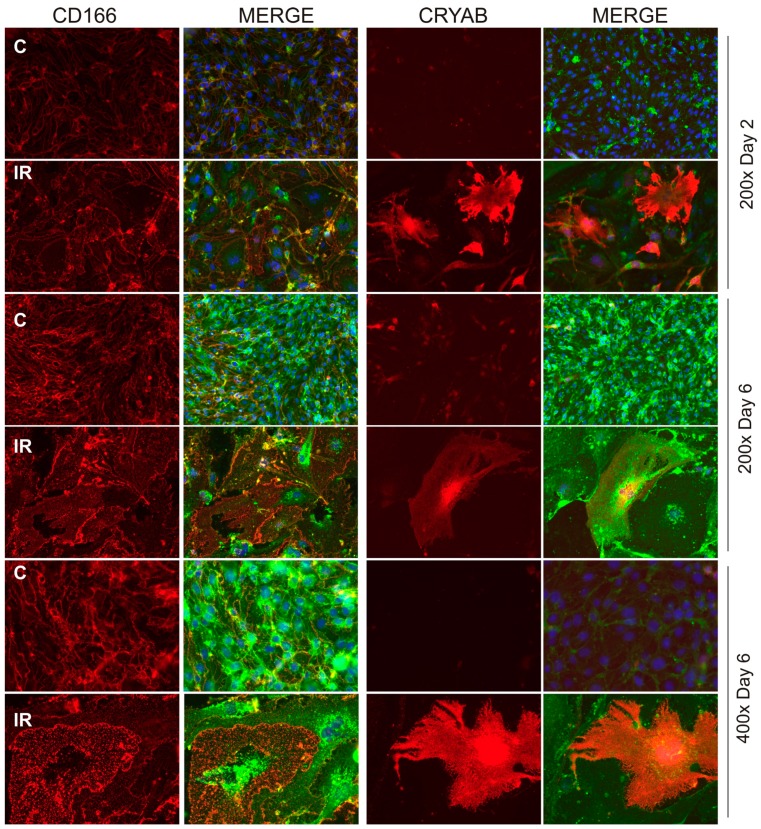
Immunofluorescent localization of target proteins in brain microvascular endothelial cells. Microvascular bEnd.3 cells were exposed to ionizing radiation (20 Gy) or sham treatment in 8-well chamber slides and immunostaining performed after 2 or 6 days on fixed (2% PFA, 5 min) but non-permeabilized cells. Representative immunofluorescent images of bEnd.3 cells probed with anti-CD166 or anti-CRYAB antibodies. Irradiated cells were typically senescent, showing a considerably enlarged, hypertrophic cell type with multi-lobed or fragmented nuclei. Target protein (AF647, red); wheat germ agglutinin surface marker (AF488, green); nuclei were stained with DAPI (blue). Original magnification, 200× or 400×, as indicted.

We further investigated the effect of radiation on CD166 and CRYAB expression using cultured brain microvascular endothelial cells to gain a better understanding of subcellular localization. Cells were examined after irradiation or sham treatment over a period of 6 days by fixing briefly in para-formaldehyde without permeation, to examine surface expression by immunostaining and fluorescence microscopy (Figure 2). Cells that remained adherent after radiation progressively developed a characteristic senescent morphology and phenotype, including flattening and enlargement, as previously described and characterized in this cell type [14]. Consistent with the in vivo findings, CD166 was present on non-irradiated cells, where it localized predominantly at the intercellular junctions between cells (Figure 2). In response to radiation, CD166 translocated from the intercellular junctions and accumulated in small immune-positive puncta across the surface of the enlarged, senescent cells.

For CRYAB, expression was scarcely detected in the population of non-irradiated endothelial cells (0.5% ± 0.2% were CRYAB-positive at day 2; 0.6% ± 0.2% CRYAB-positive at day 6), but after irradiation the number of CRYAB-positive cells increased significantly (21.1% ± 9.2% by day 2; 30.8% ± 2.5% by day 6; *p* < 0.001, *n* = 3) (Figure 2). This de novo induction of CRYAB in response to radiation was consistent with the immunostaining of AVM tissue sections and demonstrated surface exposure occurring in a sub-population of the senescent-like cells that became enlarged and hypertrophic.

### 2.4. Radiation Increases CD166 and CRYAB Expression at the Apical Surface

To assess CD166 and CRYAB expression changes more quantitatively, Western analysis was used to compare total protein levels in whole cell lysates taken from brain endothelial cells with biotin-tagged, streptavidin-enriched extracts that represent only the cell surface fraction.

**Figure 3:**
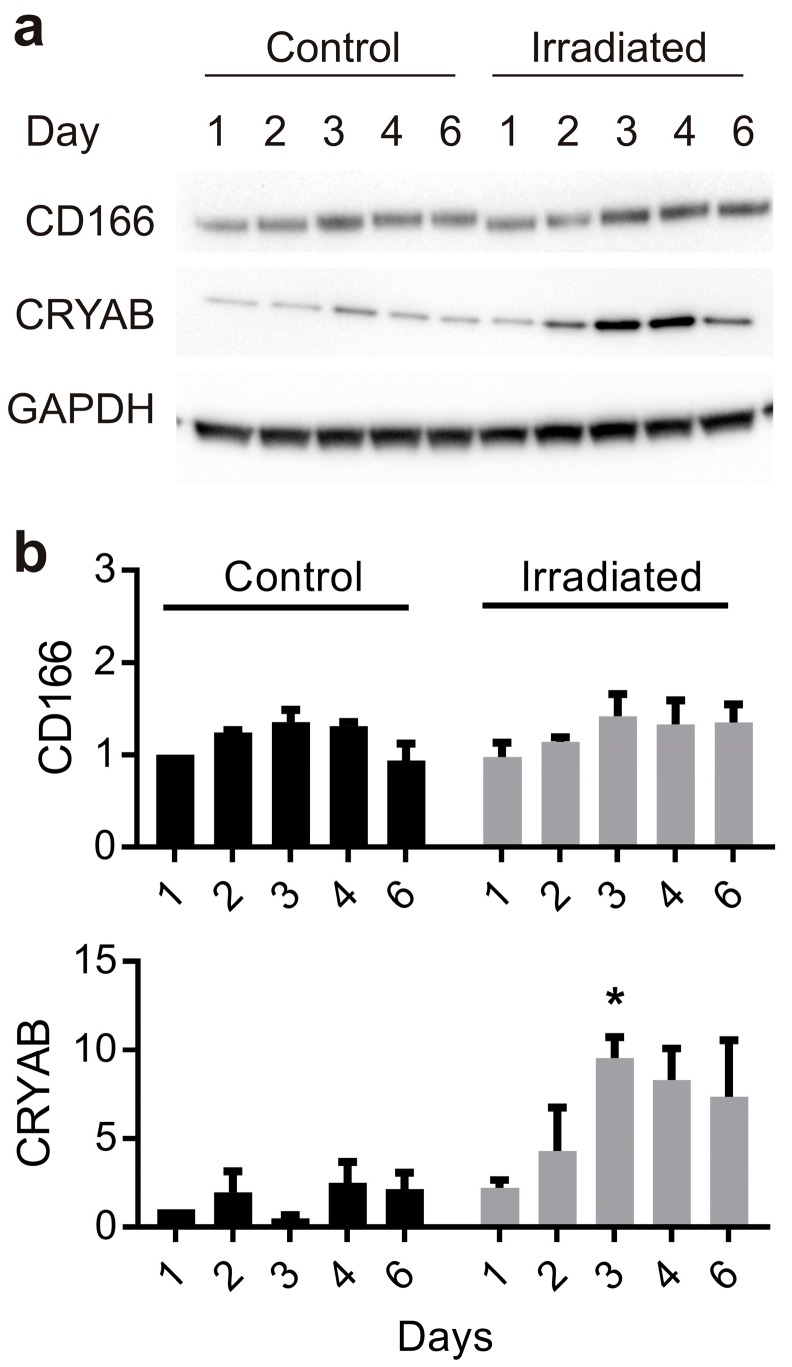
Time course of radiation-stimulated target protein expression in vitro. Representative Western immunoblots probed for CD166 and CRYAB expression in whole cell lysates (15 µg) extracted 1−6 days after sham or radiation treatment (20 Gy) of bEnd.3 cells by linear accelerator (**a**). Western blot images were quantitated using Image J and intensity, compared to respective day 1, non-irradiated controls for each blot. GAPDH was used to ensure equal loading (**b**). Data represent mean ± SEM of three independent experiments. Two-way ANOVA with Sidak’s multiple comparison test, * *p* < 0.05.

In whole cell lysates, significant basal levels of CD166 protein (105−110 kDa) were evident in non-irradiated cells, consistent with previous findings; radiation had minimal effects on total protein levels in the first 6 days (Figure 3a,b). In contrast, very low basal levels of CRYAB monomer (20 kDa) were detectable in whole cell extracts from non-irradiated cells but this increased significantly in response to radiation in a time-dependent manner (Figure 3a,b). These patterns of expression were consistent with those demonstrated by immunostaining.

**Figure 4:**
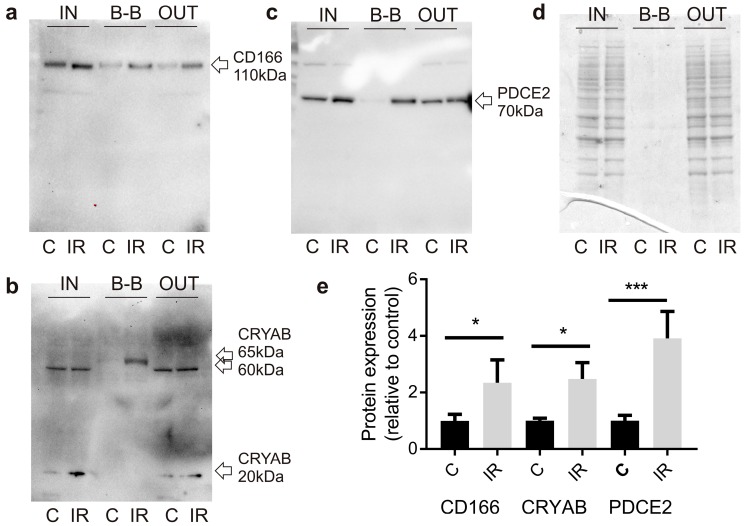
Radiation alters target expression in biotin-bound protein fractions. Representative Western immunoblot images of fractionated extracts from biotin-labeled bEnd.3 cells before and after streptavidin enrichment. Membranes were probed for CD166 (**a**), CRYAB (**b**), or PDCE2 (**c**). Representative coomassie-stained SDS-PAGE gel showing protein loadings (**d**). IN, total cell extract prior to streptavidin enrichment, 15 µg loaded; B-B, biotin-bound fraction, 30 µg loaded; OUT, non-biotinylated fraction eluted after streptavidin binding, 15 µg loaded; C, control; IR irradiated. Images are representative of 3−5 independent experiments. (**e**) Proteins in biotin-bound extracts were quantitated using Image J. Band intensities in irradiated samples were compared to band intensities in non-irradiated control samples for each blot. Paired Student’s t-test, * *p* < 0.05, *** *p* < 0.001.

Relative quantitation of surface expression was investigated by biotin-tagging proteins at the day 6 time point post-irradiation or sham, with subsequent streptavidin-enrichment of biotinylated proteins prior to Western blotting. Proteins in the biotin-tagged extracts (biotin-bound or “BB” extract) from non-irradiated and irradiated cell cultures were compared to the total protein input before streptavidin enrichment (IN), and the protein output remaining after removal of streptavidin-enriched, biotin-tagged surface proteins (OUT) (Figure 4a–e). In the biotin-bound extracts, a very different pattern of expression was observed between non-irradiated and irradiated extracts for each target relative to that observed in whole cell extracts. Consistent with the pattern of CD166 immunostaining, a significant level of CD166 expression was apparent at the surface, even in the non-irradiated, biotin-tagged extracts, however, expression increased 2-fold (*p* < 0.05) in irradiated cells in the biotin-enriched, surface fractions (Figure 4a,e). For CRYAB, whole cell extracts demonstrated the presence of strong bands consistent with the predicted size of the monomeric CRYAB protein (20 kDa) and there was significant induction in response to radiation (Figure 4b,e). A second weak band was also detected, with an approximate molecular weight of 60 kDa. However, the intensity of this band did not change with radiation treatment. In the streptavidin-enriched extracts, the 20 kDa and 60 kDa bands were completely absent, suggesting these proteins were intracellular and not present at the cell surface. Instead, a third protein band of approximately 65 kDa was repeatedly identified in the irradiated extracts (*n* = 3). This third band was not present in the INPUT samples representing total protein, suggesting the surface-localized form represents only a small fraction of the total protein pool. This band was not evident in non-irradiated extracts but increased approximately 2-fold (*p* < 0.05) in response to radiation (Figure 4e). These findings suggest that radiation induces a small pool of atypical CRYAB that is able to translocate to the cell surface.

As a positive control, the same protein extracts were analyzed for PDCE2 expression. PDCE2 is a mitochondrial protein we previously validated as undergoing an atypical surface translocation in response to radiation [5]. No band was evident in the non-irradiated, biotin-labelled extracts, but a strong band was induced approximately 4-fold (*p* < 0.001) in the irradiated, biotin-tagged protein extract (Figure 4c,e), in line with our earlier findings.

## 3. Discussion

The ability of stereotactic radiosurgery to deliver a highly focused dose of radiation to a designated region provides a potential priming mechanism for the induction of novel biomarkers. Radiation is known to induce a myriad of cellular and molecular changes, depending on dose and cell type. Studying changes that occur specifically at the cell surface increases our understanding of how radiation-damaged cells may interact with their local environment after exposure, but could also provide a pool of targets for ligand or antibody-directed drug delivery in pathologies amenable to stereotactic radiosurgery, such as brain AVMs.

For target discovery, we employed a combined approach of surface protein biotinylation with proteomic analysis, as we require target localization specifically at the luminal endothelial surface. Using this approach, we previously identified and validated several novel proteins that demonstrate non-canonical surface translocations in response to radiation [5,14]. Here, we extended our proteomic discovery approach and identified and validated two additional proteins for their endothelial expression and surface translocation in response to radiation.

CD166 demonstrated a mean 3-fold increase in response to radiation in the proteomic datasets, but was also of interest as it has previously been investigated as a vascular target in prostate cancer and an internalizing, single-chain variable fragment (scFV) antibody to CD166 developed [19]. Using Western blotting, we observed a steady level of expression of the CD166 protein in non-irradiated cells both in vitro and in vivo that did not change significantly in response to radiation, although there was a small increase over time in both irradiated and non-irradiated cells, most likely due to ongoing adjustments in cell confluence and the nature of cell–cell contacts. However, there was a significant increase in the fraction at the apical surface that could be identified after biotin-labeling and enrichment. Most notably, a significant redistribution of CD166 across the apical surface of the enlarged senescent cells was observed using immunofluorescence, consistent with this finding. This effect of radiation on CD166 was quite different to those observed for other inflammatory adhesion molecules, such as intercellular adhesion molecule 1 (ICAM-1) and vascular cell adhesion molecule 1 (VCAM-1), where surface exposure was regulated primarily at the level of transcription [7]. Like ICAM-1 and VCAM-1, CD166 can play a role at the apical surface in transendothelial monocyte migration but is primarily localized on epithelial and endothelial cells at intercellular junctions, forming an adhesive complex between cells that helps to maintain tissue architecture [18]. Thus, radiation-induced changes to CD166 localization may contribute to the pro-inflammatory milieu by increasing monocyte binding, as well as extravasation through disrupted junctions. As a molecular target, further experiments are required to assess whether the level of induction in response to radiation can overcome the relatively high basal levels of exposure of CD166 on the vasculature to give the necessary specificity required for drug delivery, but the results suggest that radiosurgery could potentially be investigated in other contexts, such as prostate cancer, for further enhancement of CD166 expression and improved targeting specificity [12].

CRYAB was chosen for further investigation in this study, due to its presence in all three proteomic datasets analyzed here with the animal model, but also because independent biotinylation and proteomic studies in cultured cells showed a 7-fold (*p* < 0.001) increase in surface expression after radiation, although it was not independently validated at the time [14]. CRYAB is a member of the small heat shock family of proteins that acts as a molecular chaperone to misfolded proteins, maintaining them in large soluble aggregates. It is well-known to be up-regulated in neurodegenerative proteinopathies such as Alzheimer’s disease and Parkinson’s disease [24] and has been identified as a candidate autoantigen in multiple sclerosis [22,25]. CRYAB is up-regulated in response to various stresses, including radiation, and plays a role in cellular survival and radioresistance [20,21,26]. In the vasculature, CRYAB is expressed in a subset of human tumor vessels, but not in normal capillaries, and is thought to assist tumor angiogenesis as it promotes survival [27,28]. In accordance with the literature, we observed negligible CRYAB expression in the normal vasculature but demonstrated a significant induction at the endothelium in response to radiation. Western blotting demonstrated that the majority of the protein appeared to remain intracellular, however, a novel 65 kDa immunopositive band was detectable in the biotin-labeled fraction, that was consistently identified in three independent experiments. The absence of this band in the whole cell extracts suggests this form of CRYAB may represent a very small proportion of the total CRYAB pool, but its presence only in response to radiation suggests a potentially unique radiation target.

It is unclear at this time what form of CRYAB this band may represent or whether it has a unique function. CRYAB is typically cytoplasmic but can translocate to the mitochondria during heat shock, where it acts as an inhibitor of apoptosis [27], and there is evidence for its secretion in exosomes in certain cell types [26,29,30]. Cubedo et al., identified the presence of both monomeric (20 kDa) and oligomeric (36 and 50 kDa) CRYAB forms in ischemic cardiomyoctes but found only the larger oligomeric forms could be isolated in the insoluble membrane fraction associated with exosomal translocation and secretion [26]. Fujii et al., demonstrated that very high doses ( > 1000 Gy) of gamma radiation delivered directly to α-crystallin could induce cross-linking and molecular weight modifications [31]. While this dose is far higher than the one used in the current study, the relatively small fraction of modified CRYAB could fit with the hypothesis that radiation produces a cross-linked multimeric form, but further investigation is required to fully analyze the nature of this isoform and its uniqueness in the vasculature for targeting.

As per our previous study, the proteomic aspect of this study was limited by the necessity to combine multiple animal AVM extracts into one for analysis at each time point, given the unanticipated low level of proteins that were obtained after enrichment [5]. This was not ideal, and future experiments of this nature would be advised to increase animal numbers in anticipation of this. However, we consider it a viable discovery approach, assuming downstream validation is performed as for any “omics” approach. The advantage of the present study was the availability of data from multiple time points, *in lieu* of animal replicates, which gave us greater confidence in the preliminary “hits” as potential targets. Inclusion of a non-AVM vessel extraction would also have been of value to assess basal levels of protein relative to the sham AVM and irradiated AVM samples, as the surgical creation of the AVM may also have altered basal levels of protein expression. Despite this, considering that this is our third proteomic study to independently identify the mitochondrial protein PDCE2 as a radiation-stimulated surface protein [5,14], this further supports the approach as a consistent and valid method for investigation of the surface proteome. Validation of other identified proteins in the proteomic datasets may be of interest to clarify any functional role in radiation injury or potential utility for vascular targeting. Further assessment of these molecules with respect to their internalization or maintenance at the cell surface would also be of value for assessing potential downstream applications. For vascular targeting with thrombin conjugates, prolonged surface exposure is desirable to allow contact with blood components for coagulation; in contrast, internalization and lysosomal activation is useful for cancer targeting.

Future studies will use in vivo imaging approaches to further investigate CD166, CRYAB and PDCE2 biodistribution at the AVM vascular wall, however the current findings support the observations that radiation drives multiple non-canonical transport pathways regulating subcellular protein localization and luminal exposure of non-classical surface proteins and that further investigation of the transport mechanisms and functional outcomes of translocation is warranted. In addition, the findings support our hypothesis that focused radiosurgery may be a source of “neo-antigens”, suitable for ligand-directed vascular targeting in AVMs.

## 4. Materials and Methods

### 4.1. Animal Model and Gamma Knife Surgery (GKS)

All experiments involving animals were approved by the Macquarie University Animal Care and Ethics Committee (Sydney, Australia) under approval numbers 2014/013 (1 April 2014) and 2014/053 (1 January 2015). Animal experiments were performed in accordance with committee guidelines and the Australian Code of Practice for the Care and Use of Animals for Scientific Purposes. The rat AVM model is formed in Sprague–Dawley rats by surgically anastomosing the rostral end of the left jugular vein to the center of the left common carotid artery, as has been described in detail previously [5,15,16]. The arteriovenous fistula and the arterialized veins (of the model AVM) are irradiated six weeks after creation in anesthetized animals using a single-fraction stereotactic radiosurgical dose, which delivers a minimum of 20 Gy to the margin of the defined AVM, to a peak value of 40 Gy at the center. This was administered using a Leksell Gamma Knife Perfexion (Elekta Instruments, Stockholm, Sweden) at Macquarie University Hospital (Sydney, Australia), as described previously [5,15]. Sham-irradiated animals were treated identically but did not receive any radiation.

### 4.2. Tissue Biotinylation and Proteomic Analysis

A cohort of 24 animals with AVM creation (*n* = 12 sham, *n* = 12 GKS) were used for in vivo biotin-labeling of luminal surface proteins. In vivo biotinylation with membrane-impermeable EZ-link Sulfo-NHS-LC Biotin (Thermoscientific, Waltham, MA, USA), and subsequent decellularization and protein extraction, was performed as described previously [5]; however, for this study the concentration of sodium dodecyl sulphate in the extraction buffer was reduced 10-fold from 1% to 0.1% to reduce carryover and interference in downstream mass spectrometry. Biotin labeling was performed either at day 3 (*n* = 8), day 7 (*n* = 8) or day 21 (*n* = 8) post-irradiation or sham treatment (*n* = 4 per treatment group, per time point). However, as initial extracts obtained from single AVM tissues contained only limited amounts of protein, it was necessary to pool the four available tissue samples per treatment group and time point to increase the detection limit. Enrichment of the biotin-bound proteins in the final six pooled samples with Streptavidin–Sepharose HP (GE Healthcare, Pittsburgh, PA, USA) and downstream proteomic sample analysis, using data-dependent and SWATH LC/MS/MS procedures [32], was as described [5,14].

### 4.3. Cell Culture and Western Analysis

Cultured mouse brain microvascular endothelial cells (bEnd.3; ATCC CRL-2299) were irradiated by linear accelerator (Elekta Synergy, Crawley, UK) (20 Gy) at Macquarie University Hospital (Sydney, Australia), as previously described [5,14]. At designated time points, cells were immunostained to examine protein localization, or whole cell lysates were extracted for western analysis of total protein content [5,14]. Alternatively, bEnd.3 cells were biotinylated in vitro and whole cell extracts subjected to streptavidin-enrichment of surface-accessible proteins before SDS-PAGE and Western analysis as per published methods [5,14].

### 4.4. Immunohistochemistry and Immunocytochemistry

A second cohort of rats received AVM creation surgery and irradiation (*n* = 3) or treatment (*n* = 3) as described above but without biotinylation. At 3 days post-irradiation or sham treatment, these animals were perfused with 500 mL phosphate-buffered saline (PBS, pH 7.4) and the excised AVM tissue snap frozen in OCT freezing compound (ProSciTech, Thuringowa Central, Australia) and used for immunohistochemistry. Cryosections (10 µm) of non-biotinylated AVM tissues were fixed with 4% paraformaldehyde for 20 min and processed as described previously [5]. Immunocytochemistry was performed on irradiated bEnd.3 cells fixed with 4% paraformaldehyde for 5 min without cell permeabilization to enable assessment of surface localization [5,14].

Antibodies against CRYAB (mouse monoclonal, ab13496, Abcam, Cambridge, MA, USA), and CD166 (goat polyclonal, PA5-47083, Thermo Fisher Scientific) were used with species-specific AlexaFluor647-conjugated secondary antibodies (Life Technologies, Grand Island, NY, USA). Ex vivo sections stained with CD166 were co-stained with anti-CD31 mouse monoclonal antibody (#550025 PECAM-1; BD Biosciences, San Jose, CA, USA) and species-specific AlexaFluor488-conjugated secondary antibody (Life Technologies) to visualize the endothelium. Control sections incubated with rabbit IgG (Santa Cruz Biotechnologies, Dallas, TX, USA) or mouse IgG (BD Biosciences) showed no reactivity. Antibodies against PDCE2 (rabbit polyclonal, sc-32925, Santa Cruz Biotechnologies) were used as a positive control in Western analyses. For immunofluorescence, cells were co-stained with an AF488-labelled lectin, wheat germ agglutinin (Life Technologies), to highlight cell surface area. Nuclei were counterstained with 4′,6-diamidino-2-phenylindole dihydrochloride (DAPI, 5 μg/mL, Life Technologies). Digital images were captured under fixed parameters using a Zeiss microscope with AxioCam HRc camera and Zen 2012 software (Carl Zeiss Microscopy, Jena, Germany).

### 4.5. Statistical Analysis

All statistical analyses were performed with Prism version 7.02 (GraphPad Software, La Jolla, CA, USA). For Western blotting, a two-sided, paired Student’s t-test was used to compare relative protein expression between biotin-enriched extracts; multiple comparisons were performed using two-way ANOVA with Sidak’s post-hoc analysis. Two-sided, unpaired Student’s *t*-tests were used to compare immunostaining data. Data are shown as mean ± SEM. Statistical significance was set at *p* < 0.05.

## Figures and Tables

**Table 1:** Proteins from the proteomic datasets that increased in response to radiation at all time points.

Uniprot Accession Number|Protein Symbol	Fold-Change at Time Point (Days)			Protein Name/Alternate Name
	3	7	21	
O35112|CD166	3.5	1.4	4.0	Activated leukocyte cell adhesion molecule (ALCAM)
Q09073|ADT2	1.8	1.4	2.7	ADP/ATP translocase 2 (SLC25a5)
B0BNL4|HRG1	1.6	8.1	1.6	Heme responsive gene 1 (SLC48a1)
P23928|CRYAB	2.0	1.5	1.6	αB-crystallin/heat shock protein β5 (HSPB5)
P08461|ODP2	2.5	2.7	1.6	Dihydrolipoamide S-acetyltransferase (DLAT) or pyruvate dehydrogenase complex subunit E2 (PDCE2)

## Supplementary Materials

Supplementary materials can be found at https://www.mdpi.com/1422-0067/20/23/5830/s1.
