# Supplementary material for: Radiation-Stimulated Translocation of CD166 and CRYAB to the Endothelial Surface Provides Potential Vascular Targets on Irradiated Brain Arteriovenous Malformations
Source: Int J Mol Sci. 2019 Nov 20;20(23):5830. doi: 10.3390/ijms20235830 (PMC6929092; doi:10.3390/ijms20235830)
Supplement: Supplementary file 1 [file ijms-20-05830-s001.pdf]

Supplementary Material: International Journal of Molecular Sciences

Radiation-stimulated translocation of CD166 and CRYAB to the endothelial surface provides potential vascular targets on irradiated brain arteriovenous malformations

Lucinda S. McRobb[a*], Matthew J. McKay[b], Andrew J. Gauden[a], Vivienne S. Lee[a], Sinduja Subramanian[a], Santhosh George Thomas[a], Markus K.H. Wiedmann[a], Vaughan Moutrie[c], Michael Grace[c], Zhenjun Zhao[a], Mark P. Molloy[b], Marcus A. Stoodley[a]

Corresponding author: lucinda.mcrobb@mq.edu.au

Supplementary Table S1: Proteins (rat) identified in SWATH proteomic analysis with fold-change ≥ 1.4 at 3 weeks post-radiation.

		Fold-change (irradiated/sham)		
**Accession #**	**Protein**	**Day 3**	**Day 7**	**Day 21**
Q6NYB7	Q6NYB7|RAB1A	1.4	0.4	14.8
Q63081	Q63081|PDIA6	0.5	1.2	7.6
P09006	P09006|SPA3N	0.9	9.7	6.8
Q91Y81	Q91Y81|SEPT2	1.3	1.1	6.5
P62250	P62250|RS16	1.8	0.6	6.5
Q2TL32	Q2TL32|UBR4	0.2	1.1	6.1
P11507	P11507|AT2A2	1.0	0.4	6.0
P28480	P28480|TCPA	0.1	0.8	4.8
P62832	P62832|RL23	5.3	0.6	4.3
P11497	P11497|ACACA	1.7	1.0	4.2
Q29RW1	Q29RW1|MYH4	0.8	1.6	4.2
**O35112**	**O35112|CD166**	**3.5**	**1.4**	**4.0**
P06399	P06399|FIBA	0.4	0.6	4.0
P05712	P05712|RAB2A	0.8	1.3	3.9
P08649	P08649|CO4	1.1	0.8	3.5
P54290	P54290|CA2D1	0.5	3.3	2.8
**Q09073**	**Q09073|ADT2**	**1.8**	**1.4**	**2.7**
Q2PQA9	Q2PQA9|KINH	2.9	0.3	2.5
P05371	P05371|CLUS	1.2	0.7	2.5
P62246	P62246|RS15A	0.2	0.7	2.5
Q6YAT4	Q6YAT4|SGCE	0.1	2.1	2.4
Q6IG02	Q6IG02|K22E	0.5	0.2	2.4
Q6AXT5	Q6AXT5|RAB21	0.5	0.6	2.4
Q8VHF5	Q8VHF5|CISY	1.3	1.1	2.2
Q01177	Q01177|PLMN	1.0	0.9	2.2
Q9ERH3	Q9ERH3|WDR7	0.8	0.7	2.2
P70490	P70490|MFGM	0.7	0.9	2.1
P61980	P61980|HNRPK	0.9	0.9	2.1
P26284	P26284|ODPA	0.8	0.8	2.1

Q6P7B0	Q6P7B0 | SYWC	0.8	0.6	2.1
P17475	P17475 | A1AT	0.6	0.8	2.0
P04642	P04642 | LDHA	0.8	1.3	2.0
P62718	P62718 | RL18A	0.5	5.2	1.9
P62268	P62268 | RS23	0.7	1.7	1.9
P04636	P04636 | MDHM	0.6	0.7	1.9
P00507	P00507 | AATM	0.8	1.9	1.9
P35565	P35565 | CALX	0.8	1.2	1.9
Q642A6	Q642A6 | VWA1	0.8	0.6	1.8
P14480	P14480 | FIBB	1.3	0.7	1.8
O35763	O35763 | MOES	0.9	0.4	1.8
Q5XIF6	Q5XIF6 | TBA4A	1.1	0.8	1.8
P07895	P07895 | SODM	0.7	1.2	1.8
P62914	P62914 | RL11	1.6	0.5	1.8
P23514	P23514 | COPB	1.2	1.0	1.7
P16391	P16391 | HA12	1.2	0.1	1.7
Q63041	Q63041 | A1M	0.6	0.7	1.7
Q63617	Q63617 | HYOU1	0.6	1.3	1.7
**B0BNL4**	**B0BNL4 | HRG1**	**1.6**	**8.1**	**1.6**
**P23928**	**P23928 | CRYAB**	**2.0**	**1.5**	**1.6**
P02680	P02680 | FIBG	0.7	0.6	1.6
P12346	P12346 | TRFE	0.7	0.7	1.6
Q07009	Q07009 | CAN2	1.4	0.8	1.6
**P08461**	**P08461 | ODP2**	**2.5**	**2.7**	**1.6**
Q6AXV4	Q6AXV4 | SAM50	0.9	0.8	1.6
P14046	P14046 | A1I3	0.7	1.0	1.6
P01946	P01946 | HBA	0.5	1.0	1.6
P41350	P41350 | CAV1	1.0	1.8	1.5
P04276	P04276 | VTDB	0.8	0.7	1.5
Q4AEF8	Q4AEF8 | COPG1	1.0	0.8	1.5
Q9Z2L0	Q9Z2L0 | VDAC1	1.3	0.4	1.5
P11980	P11980 | KPYM	0.9	1.0	1.5
P04937	P04937 | FINC	0.6	0.6	1.5
P15800	P15800 | LAMB2	1.2	0.7	1.5
Q5BK63	Q5BK63 | NDUA9	1.2	8.1	1.5
P02770	P02770 | ALBU	0.6	1.0	1.5
Q9R1Z0	Q9R1Z0 | VDAC3	0.7	1.2	1.4
P09650	P09650 | MCPT1	1.6	0.9	1.4
Q64122	Q64122 | MYL9	0.9	0.8	1.4
P20059	P20059 | HEMO	0.6	0.7	1.4
P50878	P50878 | RL4	1.3	0.2	1.4
Q62930	Q62930 | CO9	1.3	0.9	1.4
Q05030	Q05030 | PGFRB	0.2	1.9	1.4
